# Investigation of biological activities of plant extract and green synthesis silver nanoparticles obtained from Gilaburu (*Viburnum opulus* L.) fruits

**DOI:** 10.3906/kim-2108-48

**Published:** 2021-10-17

**Authors:** Birgütay ŞAHİN, Ali Savaş BÜLBÜL, İ. Seyfettin ÇELİK, Nesrin KORKMAZ, Ahmet KARADAĞ

**Affiliations:** 1 Department of Molecular Biology and Genetics, Faculty of Science, Bartın University, Bartın, Turkey; 2 Department of Emergency Aid and Disaster Management, Faculty of Applied Sciences, Bayburt University, Bayburt, Turkey; 3 Department of Medical Pathology, Faculty of Medicine, Kahramanmaraş Sütçü İmam University, Turkey; 4 Department of Basic Sciences and Health, Hemp Research Institute, Yozgat Bozok University, Yozgat, Turkey; 5 Department of Chemistry, Faculty of Arts and Sciences, Yozgat Bozok University, Yozgat, Turkey

**Keywords:** Biological activity, fruit extract, green synthesis, HUVEC (human umbilical vein endothelial cells), MCF-7 (human breast cancer), *Viburnum opulus* L

## Abstract

In this study, biological activities of silver nanoparticles (AgNP) synthesized by green synthesis method using the fruit extract of Gilaburu (*Viburnum opulus* L.) plant were investigated. The characterization of the synthesized AgNPs was performed by ultraviolet visible region spectroscopy (UV-Vis), Fourier transform - infrared spectroscopy (FTIR), scanning electron microscopy (SEM), and X-ray diffraction (XRD) techniques. The crystal size was found to be 52.32 nm when the XRD data were calculated with the Debye-Scherrer’s equation. The antimicrobial activities of extract and AgNPs were investigated by microdilution and disk diffusion methods. Antibiofilm activities were examined by the crystal violet technique. The cytotoxic effects of the extract and AgNPs against MCF-7(human breast cancer) and HUVEC (human umbilical vein endothelial cells) cell lines were evaluated by MTT assay. The IC50 valuesfor the HUVEC line were found to be 0.97 mg/mL (AgNP) and 85.24 mg/mL (extract), while the IC50 values for the MCF-7 line were determined as 0.022 μg/mL (AgNP) and 0.021 μg/mL (extract). To our knowledge, this is the first report to comprehensively examine the biological activities of *V. opulus* L. extract and biosynthetic AgNPs.

## 1. Introduction

Approximately 25%–48% of the drugs produced today consist of plants or their synthetic derivatives [1]. Phytochemicalsare plant derivatives, and more than 10,000 of them are used as anti-cancer compounds in cancer treatment. It has also been shown that they can increase the effectiveness of anti-cancer compounds and reduce their toxic effects [2].

Another rapidly developing field of study in recent years is the green synthesis method for the reduction of metal salts through plants containing phytochemicals. This field, which is one of the sub-branches of nanotechnology, has begun to be used in many areas such as the production of materials or drugs for cancer treatment, the prevention of microbial diseases or their treatment. The most important feature of this method is that it can be used as a very cheap and environmentally friendly technique compared to other nanotechnological methods.

Thanks to the physical and chemical technologies that have been used in nanoparticle production for a long time, high resolution, desired small particles can be produced in a short time, but more new technologies should be investigated due to their high toxic content, poor particle stability, and expensive technologies [3,4]. In this sense, the examination of substances and living things that already exist in nature, containing perfectly designed nano-dimensions, has inspired scientists, and the production of inorganic substances using living structures has begun to be investigated. As a result of researches, the term “green nanotechnology” has emerged, which is environmentally friendly technology low in toxic substance content and based on the production of nanoparticles from living cells. This term is used for working methods that reduce the problem of waste products, that are not harmful to human health, and that are easily applicable, within the scope of nanotechnology [5–7]. Especially green plant extracts and microorganisms are used within the scope of green nanotechnology. Although many living organisms are used in this sense, *Aloe vera, Azadirachta indica* (linden), *Camellia sinensis* (tea), *Jatropha curcas* (nutmeg), *Acalypha indica* (indian nettle) can be given as examples [8].

Various methods are used to obtain silver nanoparticles (AgNP). Among these methods, the biological method is preferred more than other (physical and chemical) methods because it is environmentally friendly and economical [9–16]. AgNPs obtained by green synthesis are also important for living applications with their biocompatibility feature [17–20].

Gilaburu plant (*Viburnum opulus* L.) belongs to the Caprifoliaceae family, widely grown in Eastern Europe, Northern Asia and Africa. The red fruits of this species, which are generally used as an ornamental plant, are edible. The phytochemical compounds in the fruits are used in traditional medicine for the regulation of blood pressure, the treatment of cramps, the treatment of many diseases such as diabetes, digestive problems, and colds [21,22]. When scientific studies conducted in the United States and England were examined, it was observed that the consumption of 250 mL of V. opulus L. fruit juice per day has positive effects in reducing some tumors, thanks to the antioxidant compounds it contains. It has also been reported to have sedative, skeletal and muscle relaxant, vasodilation regulating, and heart-strengthening effects. It has been reported that it relaxes the vascular system, prevents constipation and is highly effective and curative against urinary difficulties and burns [23]. The main reason why the effect of V. opulus fruit on human health is high is the high anthocyanins it contains. Anthocyanins are known to have anti-cancer effects. In an experiment conducted on cells causing brain cancer, it was revealed that this molecule does not harm healthy cells and that it has a highly selective lethal role in cancer cells [24]. In general, although anthocyanins are effective on a variety of microbes, gram-positive bacteria have been shown to be more sensitive to anthocyanins than gram-negative bacteria. It is thought that the antimicrobial activity of fruits containing anthocyanins is due to the mixture of organic compounds such as anthocyanins, weak organic acids, phenolic acids, and other chemical forms, possibly due to various mechanisms and synergistic effects [25]. Especially the high anthocyanin content in V. opulus fruit pulp plays an important role in reducing silver ions and stabilizing the formed nanoparticles [26].

Within the scope of our study, silver nanoparticles (AgNP) were produced by green synthesis method using V. opulus fruit extract in the first stage. The characterization processes of the synthesized AgNPs were performed by UV-Vis spectrophotometer, scanning electron microscopy (SEM), Fourier transform infrared spectroscopy (FTIR) and X-ray diffraction (XRD) techniques. In the next stage, it is aimed to examine the biological activities (antibacterial, antibiofilm and anticancer properties) of the plant extract and AgNPs.

## 2. Materials and methods

### 2.1. Obtaining Gilaburu (*V*. *opulus*) fruit extract

The fruits of Gilaburu (*V. opulus*) were collected from Çakırkadı Village of Central district of Bartın Province during August 2020 (latitude 41° 25′ North latitude, 32° 15′ West longitude) and frozen and stored at −18°C. 35 g of ground fruit was obtained by Soxhlet extraction method in 350 mL distilled water medium. The extract in the solution was concentrated under low pressure at 40 °C with the aid of the evaporator B480 (Buchi Labortechnik AG, Flawil, Switzerland). The obtained fruit extract was stored in the refrigerator at 4 °C.

### 2.2. Green synthesis of silver nanoparticles (AgNP)

First, 100 mL of 10 mM silver nitrate (AgNO_3_) solution was prepared. 90 mL of the prepared AgNO_3_ solution was taken and added to 10 mL of *V. opulus* fruit extract on a magnetic stirrer at 65 °C. The reduction of silver ions was determined by the color change that occurred in the solution. Purification was carried out by centrifuging the solution at 15,000 rpm for 10 min. The resulting AgNPs were left to dry by removing the supernatant and washing it with distilled water twice [27,28].

### 2.3. Characterization of synthesized AgNPs

Characterization processes of AgNPs were carried out by UV-Vis, SEM, XRD, and FTIR techniques. MALA3 XMU TESCAN device was used for SEM analysis. The prepared AgNP solution was placed on a carbon-coated copper grid and images were acquired. The crystal structure of AgNPs was obtained using high-resolution XRD (SMARTLAB RIGAKU). Thermo Scientific Multiskan GO was used to take spectroscopic measurements that were made in the 300 to 800 nm range in UV-Vis analysis, which exhibited an absorption band of around 400–450 nm for AgNPs [24]. After the synthesis of AgNPs, FTIR analysis was performed to identify the different functional groups on AgNPs. Samples were detected with an infrared source with a spectrum scanned in the 400–4000 cm^−1^ range to obtain a good signal-to-noise ratio.

### 2.4. Antimicrobial activity

#### 2.4.1. Disk diffusion method

Antimicrobial activity tests of AgNPs and fruit extracts were performed by Kirby–Bauer disk diffusion method [29]. A total of 15 microorganisms consisting of 6 g positive and 9 g negative bacteria, were used in the study. Disk impregnated with *V. opulus* extract and silver nanoparticles (AgNP) as well as a ready antibiotic disk containing their positive controls Tetracycline (TE: 20mg/mL) were used for biological activity. Microorganisms were incubated in Luria Bertoni (LB) broth for 18 hours. Bacterial strains that achieved sufficient growth were inoculated with the help of sterile swaps on the surface of Petri dishes containing Nutrient Agar disk containing AgNP and plant extract were placed in petri dishes and incubated for 18–24 h at 37°C. Finally, the zone diameters were measured, and the arithmetic means of the results were taken. The experiment was run twice.

#### 2.4.2. Minimum inhibition concentration (MIC)

The antimicrobial activities of *V. opulus* extract and AgNPs were determined by minimum inhibitory concentration (MIC) and minimal bactericidal concentration (MBC) methods. For this purpose, gram-positive and gram-negative bacteria were incubated overnight for the antimicrobial activities of the synthesized AgNPs. Ninetysix-microplate wells were used for the microdilution method. LB Broth medium for bacteria was added to the wells of 96 microplates in applications carried out with the microdilution method. After preparing a series of dilutions from the adjusted concentrations of *V. opulus* extract and AgNPs, their solutions were added to the microplates and diluted. Then, a certain amount of microorganism solutions, prepared and adjusted according to 0.5 McFarland, were added to the microplates and incubated overnight at 37 °C. The lowest concentration without growth after incubation was determined as the MIC value.

#### 2.4.3. Minimum bactericidal concentration (MBC)

After determining the minimum inhibitory concentrations (MIC) of the plant extract and AgNP, the minimum bactericidal concentrations (MBC) were assessed. After obtaining the MIC values, the wells in which microorganisms did not grow were determined, and the samples were taken from these wells with the help of sterile loops and inoculated into a solid medium. It was then incubated at 37 °C for 16–18 h. After that, the minimum antimicrobial agent concentration, which killed 99.9% of the bacteria of the samples inoculated into the medium, was accepted as the MBC value. Experimental studies were run in triplicate for AgNP and plant extract.

### 2.5. Antibiofilm activity

Antibiofilm properties of *V. opulus* extract and obtained AgNPs were determined using the method of Merritt et al. (2015) [30]. *Salmonella kentucky, Enterococcus faecalis* ATCC 29212, *Bacillus subtilis* DSMZ 1971 *and Escherichia coli* CFAI ATCC 25922 bacteria were used for the antibiofilm activity test. In the first stage, microorganisms were incubated at 37 °C for a total of 48 h. To dissolve the dye, 33% acetic acid was added to the wells with gram-positive bacteria, and 95% ethanol was added to the wells with gram-negative bacteria. Microplates containing the dissolved dye were read at 600 nm by the spectrophotometer. The antibiofilm effect was calculated by comparing it with the positive control according to the formula below.


$$\text{Inhibition }\%=({\text{A}}_{\text{c}}-{\text{A}}_{\text{s}}/{\text{A}}_{\text{c}})\times 100$$

A_c_: The absorbance value of the control group at 600 nm.
A_s_: Absorbance value of the sample group at 600 nm.

Statistical analyses were made using the IBM SPSS Statistics 23.0 (SPSS Inc; Chicago, IL, USA) program. Mean, standard deviation, and percentage distributions were presented. The plant extract and AgNP antibiofilm formation efficiency data obtained from the study were interpreted by performing Pearson correlation analysis. A p value of ≤0.05 and ≤0.01 was considered to be statistically significant.

### 2.6. Anticancer activity

#### 2.6.1. Replication of cells

HUVEC (human umbilical vein endothelial cells) and MCF-7 (human breast adenocarcinoma) cell lines were placed in a medium containing 10% FBS, 1% insulin, 1% penicillin/streptomycin and kept in a 5% CO2 incubator at 37 °C for proliferation. Cells were counted by staining with trypan blue to evaluate whether the cells were proliferating adequately and cell viability. Cells that reached sufficient numbers were seeded in 96-well microplate with approximately 5000 cells in each well.

#### 2.6.2. Treatment with plant extract and AgNP

*V. opulus* extract and different concentrations of green synthesis AgNP were added to the wells containing the cells and incubated for 24 h at 37 °C in 5% CO_2_. At the end of the incubation, the viability level of the cells was determined using the MTT method.

#### 2.6.3. MTT assay

The stock solution was prepared by dissolving MTT at 5 mg/mL in PBS. After the cells were incubated for the selected time periods with the test substances, the medium was removed from the cells. 0.1 ml of MTT working solution was added to each well and incubated in a CO_2_ incubator for 3–4 h. Then, the plates were taken, and the medium was removed and 0.1 ml of DMSO was added. Optical densities of cells in the plates were read in the ELISA device at a wavelength of 570 nm [31–33].

### 2.7. Statistical analysis

Statistical analyzes were performed using GraphPad Prism software version 8 (GraphPad Software, La Jolla, CA). Two-way ANOVA test was used to calculate statistical differences in cell viability and IC50 values. P values less than 0.005 were considered to be statistically significant.

## 3. Results and discussion

### 3.1. Characterization of synthesized AgNPs

The characterization (size, morphology, crystal structure) of AgNPs synthesized by the green synthesis method was performed with different techniques. Various analytical techniques can be applied to determine different parameters such as characterization processes using UV-visible spectroscopy (UV-Vis), Fourier transform infrared spectroscopy (FTIR), X-ray diffraction (XRD) and scanning electron microscopy (SEM) techniques.

**Figure 1:**
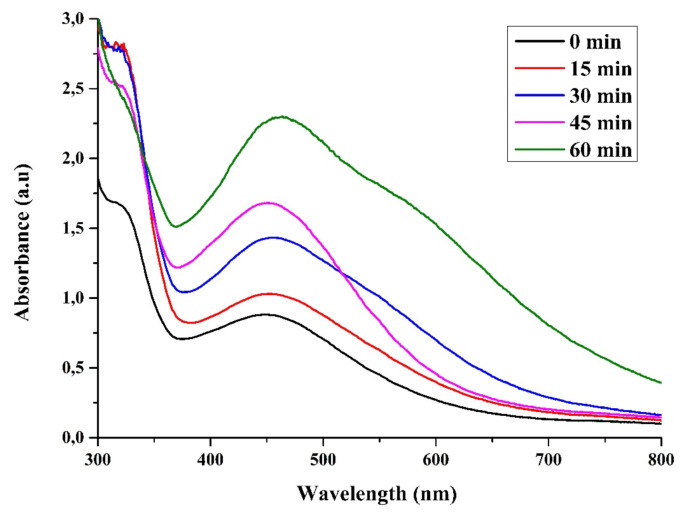
UV-Vis spectra of green synthesis silver nanoparticles (AgNP).

With the addition of fruit extract to the aqueous silver nitrate solution, a color change was observed, indicating the formation of AgNPs due to surface plasmon resonance. The absorption spectra of AgNPs formed in the reaction medium have an absorption maximum between 420 and 460 nm due to the surface plasmon resonance (SPR) of AgNPs [34]. It was observed that the silver plasmon resonance measured after 0 min by Uv-vis measurement of AgNP occurred at 420 nm, after 30 min at 442 nm, and after 1 h at 450 nm, and it increased continuously at the peak wavelength as the reaction times increased (Figure 1). This means that nanoparticles are synthesized [35–39]

**Figure 2:**
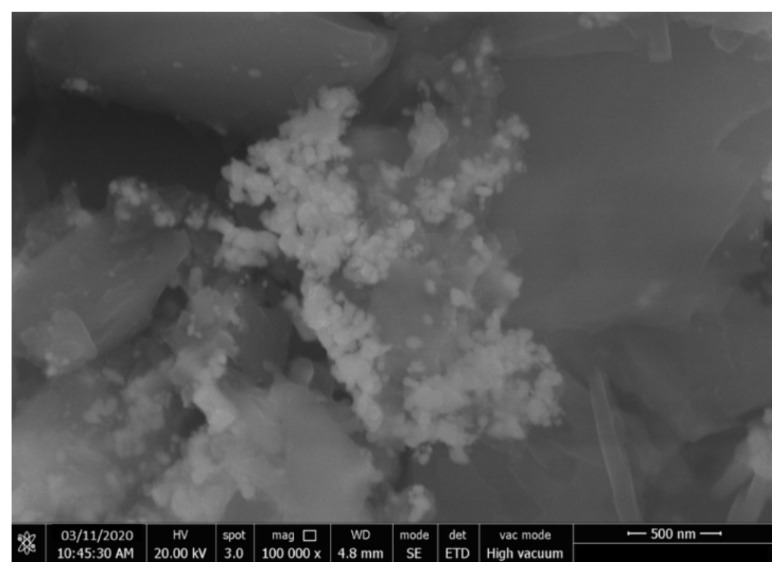
SEM images of green synthesis silver nanoparticles (AgNP).

Scanning electron microscopy (SEM) technique was used to examine the morphological properties of AgNPs obtained by biosynthesis method. SEM images of the synthesized AgNPs were taken to determine the particle size and shape of the nanoparticles. SEM images of AgNPs at 20k magnification are shown in Figure 2. The SEM image showed that the synthesized AgNPs were in a dominant spherical shape.

**Figure 3:**
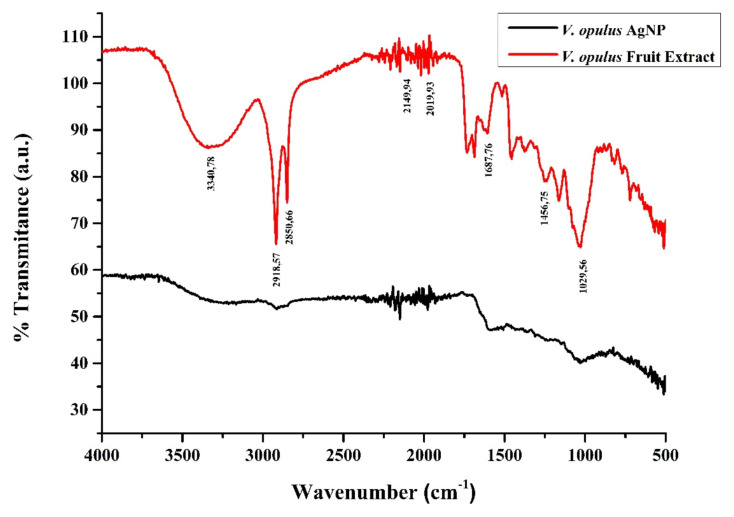
FTIR spectra of green synthesis AgNP and *V. opulus* extract.

**Figure 4:**
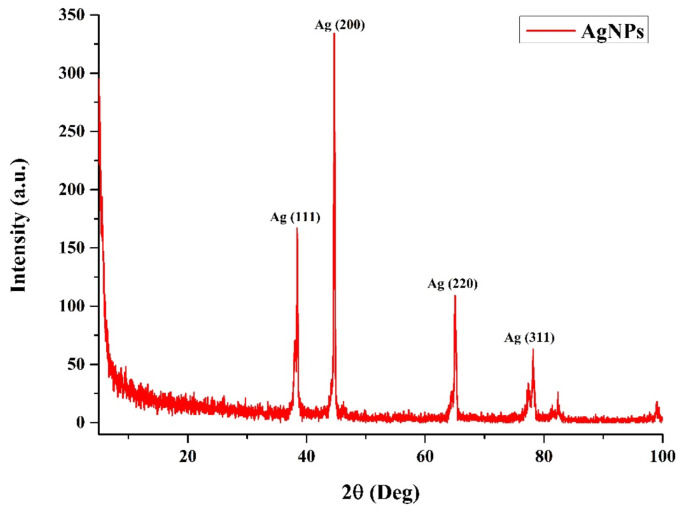
XRD image of green synthesis *V. opulus* silver nanoparticles (AgNPs).

FTIR analysis of fruit extract and AgNPs revealed the dual role of bioactive components of *V. opulus* fruits as both reducing and capping agents. When the FTIR spectra of the *V. opulus* fruit extract were examined, it was observed that bands were observed at 3340, 2918, 2850, 2149, 2019, 1687, 1456, and 1029 cm^−1^ (Figure 3). The broad absorption band at 3340 cm^−1^ corresponds to the stretching vibration of the OH (hydroxyl) in the H-linked alcohols and phenols found in the fruit extract. It was determined that the peaks observed at 2918 cm^−1^ and 2850 cm^−1^ were caused by saturated alkane (-C-H) stretching [40]. These peaks seen in the extract combined with AgNP and were observed at 2914 cm^−1^. The peaks seen at 2149 cm^−1^ and 2019 cm^−1^ showthe -N≡C bond, which is effective in the formation of peptide bonds by combining the amino acids in the structure. The vibration at 1737 cm^−1^ represents the carbonyl group. The peak at 1687 cm^−1^ corresponds to the C=C extension of alkenes originating from phenyl, and the peak at 1456 cm^−1^ corresponds to the -C-O bond of oligosaccharides. The band seen at 1029 cm^−1^ shows glucose. When the FTIR spectra of fruit extract and synthesized AgNPs are compared, it can be observed that the absorption band of the phenolic OH groups found at 3340 cm^−1^ in the fruit extract has shifted to 3420 cm^−1^ in the AgNPs spectrum. The absence of the peak of the carbonyl group indicates that the flavonoids containing these groups provide the reduction of silver, and the synthesized nanoparticles are sealed and stabilized by the same biomolecules. Four Bragg reflection peaks corresponding to the characteristic planes (111), (200), (220), and (311) of the face-centered cubic structure were observed at 2θ values of 38.44°, 44.66°, 65.01° and 78.21°. The Scherrer equation was used to calculate the average particle size on the XRD plot. The particle size was found to be 52.32 nm (Figure 4). The results are compared to previous studies that are newly introduced to the literature. There are both smaller nanoparticles than the nanoparticles we synthesized, and results are close to those obtained from our study [41–45].

### 3.2. Antimicrobial activities

Silver nanoparticles have been widely used recently based on their documented antibacterial properties [46].

#### 3.2.1. Disk diffusion method

In order to determine the antimicrobial activities of AgNPs obtained by *V. opulus* extract and green synthesis method, first the disk diffusion method was performed. In this method, disk with a diameter of 6 mm was used. The disk was impregnated with plant extracts and AgNPs to test a total of 15 microorganisms strains consisting of six gram positive bacteria (*Enterococcus faecalis* ATCC 29212, *Enterococcus durans, Listeria innocua, Staphylococcus aureus* ATCC 25923, *Staphylococcus epidermidis* DSMZ 20044, *Bacillus subtilis* DSMZ 1971) and nine gram-negative bacteria (*Salmonella enteritidis* ATCC 13075, *Salmonella typhimurium, Salmonella infantis, Salmonella kentucky, Enterobacter aerogenes* ATCC 13048, *Klebsiella pneumoniae, Pseudomonas aeruginosa* DSMZ 50071, *Escherichia coli* CFAI ATCC 25922, *Saratia marrescens* ATCC 13048). In addition, Tetracycline (TE: 10 mg/ml) antibiotic disk was used as a positive control. After the incubation period, the tetracycline antibiotic showed an inhibition zone of 7.9–19.7 mm against the test microorganisms. According to the results obtained, *V. opulus* extract *E. aerogenes* ATCC 13048, *S. infantis, K. pneumoniae, P. aeruginosa* DSMZ 50071, *S. kentucky, E. faecalis* ATCC 29212, *L. innocua, S. enteriditis* ATCC 13075, *E. durans, S. epidermidis* DSMZ 20044, *B. subtilis* DSMZ 1971 and *E. coli* CFAI ATCC 25922 showed a zone of inhibition between 7.05 and 8.15 mm against microorganisms but did not show any effect against other bacterial strains. It was found that green synthesis of AgNP showed an inhibition zone between 6.05 and 7.15 mm against *E. aerogenes* ATCC 13048, *S. infantis, K. pneumoniae, P. aeruginosa* DSMZ 50071, *S. kentucky, E. faecalis* ATCC 29212, *L. innocua, S. enteriditis* ATCC 13075, *E. durans*, that *S. epidermidis* DSMZ 20044, *B. subtilis* DSMZ 1971 and *E. coli* CFAI ATCC 25922 (Table 1).

#### 3.2.2. Minimum inhibitory concentration (MIC) and minimum bactericidal concentration (MBC) results

The minimum inhibition concentration (MIC) is defined as the lowest concentration at which there is no visible growth [47]. Fifteen bacterial strains treated with plant extracts and AgNPs in 96-well microplates were incubated for 16–18 h and then measured at 600 nm wavelength by spectrophotometer. The minimum inhibitory values (mg/mL) of *V. opulus* extract and AgNPs against the tested bacteria are given in Table 2.

Microorganism strains taken from microplates were inoculated into an antibiotic-free medium, and minimum bactericidal concentrations that ended the growth of bacteria by 99.9% were determined, and the results are shown in Table 3.

### 3.3. Antibiofilm activity results

The effects of different concentrations of *V. opulus* extract and AgNPs on antibiofilm formation on bacterial strains are evaluated in Tables 4 and 5. The applied plant extract and AgNP inhibited biofilm formation on *S. kentucky, E. faecalis* ATCC 29212, *B. subtilis* DSMZ 1971, *E. coli* CFAI ATCC 25922 strains.

As shown in Table 4, *V. opulus* extract was found to act on *S. kentucky, B. subtilis* DSMZ 1971 *and E. faecalis* ATCC 29212 bacterial strains at all concentrations whereas *E. coli* CFAI ATCC 25922 did not inhibit biofilm formation at a concentration of 6.25 mg/mL. However, the biofilm inhibition values for *V. opulus* extract at concentrations of 200 mg/mL, 100 mg/mL, 50 mg/mL, 25 mg/mL, 12.5 mg/mL, and 6.25 mg/mL were between the ranges of 44.3%–58.85%, 34.9%–52.25%, 20.05%–47.3%, 10.8%–39.6%, 5.35% and 27.35% and 8.45%–13.55%, respectively.

As shown in Table 5, *V. opulus* AgNPs had an inhibitory effect on biofilm formation on all strains applied at different concentrations. Accordingly, the biofilm inhibition values at concentrations of 1 mg/mL, 0.5 mg/mL, 0.25 mg/mL, 0.12 mg/mL, 0.06 mg/mL, and 0.03 mg/mL were between the ranges of 67.93%–86.07%, 62.77%–83.30%, 61,02%–77.06, 58.06%–71.42%, 49.19%–63.73%, and 24.74% and 54.34%, respectively.

### 3.4 Anticancer activity results

**Figure 5:**
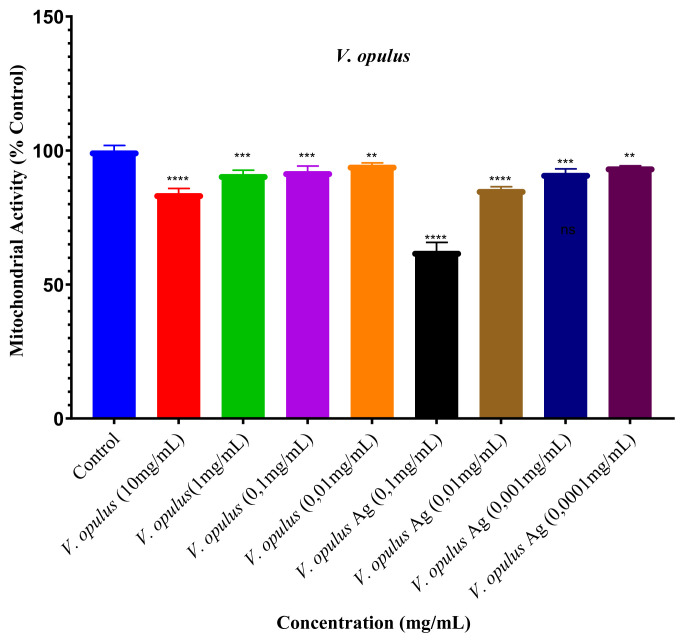
Cytotoxic effects of *V. opulus* fruit extract and green synthesis AgNP on HUVEC cell line (*: Efficacy levels of study intervals versus control group).

**Figure 6:**
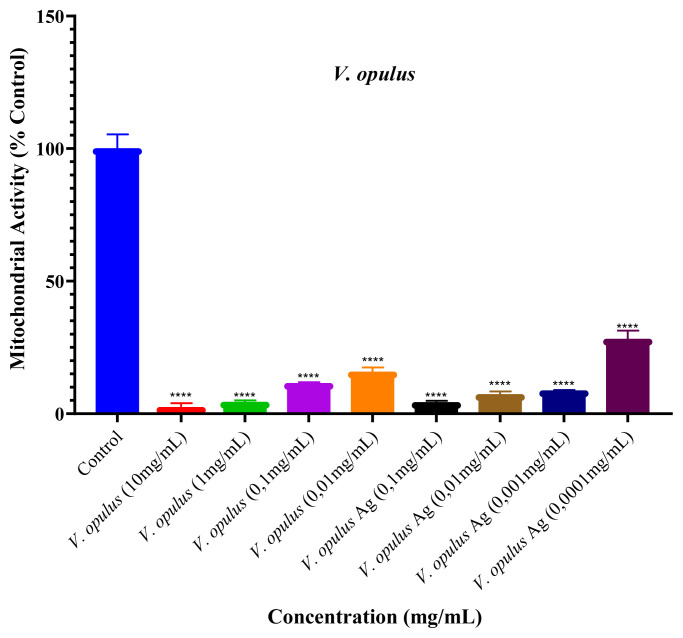
Cytotoxic effects of *V. opulus* fruit extract and green synthesis AgNP on MCF-7 cell line (*: Efficacy levels of study intervals versus control group).

The cytotoxic effects of different concentrations of *V. opulus* extract and green synthesis AgNPs (for extract: 10 mg/mL, 1 mg/mL, 0.1 mg/mL, 0.01 mg/mL; for AgNP: 0.1 mg/mL, 0.01 mg/mL, 0.001 mg/mL, 0.0001 mg/mL) on MCF-7 (human breast cancer cells) and HUVEC (human umbilical vein endothelial cells) cell lines were determined by MTT (Figures 5 and 6).

In Figure 5, the evaluation of cytotoxic effects of different concentrations of *V. opulus* fruit extract and green synthesis AgNPs on HUVEC cells used as healthy cell lines can be seen. When compared with the control group, the concentrations of 10 mg/mL, 1 mg/mL, 0.1 mg/mL (100 μg/mL) and 0.01 mg/mL (10 μg/mL) of *V. opulus* fruit extract on cell lines viability rates were found to be 84%, 91.3%, 92.3% and 94.7%, respectively. 0.1 mg/mL (100 μg/mL), 0.01 mg/mL (10 μg/mL), 0.001 mg/mL (1 μg/mL), and 0.0001 mg/mL (0.1 μg/mL) concentrations of AgNPs on cell lines as a percentage of cell viability were found as 62.5%, 85.6%, 91.7% and 94.1%, respectively. When the results obtained were assessed, the cytotoxic effect of green synthesis AgNPs on HUVEC cell lines was found to be stronger than *V. opulus* extract.

The IC_50_ value of the green synthesis AgNPs was found to be 0.97 mg/mL (970 μg/mL) for the HUVEC cell line, and the IC_50_ value of the *V. opulus* fruit extract was 85.24 mg/mL.

Figure 6 shows the evaluation of cytotoxic effects of different concentrations of *V. opulus* fruit extract and green synthesis AgNPs on MCF-7 cells used as healthy cell lines. Compared with the control group, the percentage of cell viability of *V. opulus* fruit extract on cell lines at concentrations of 10 mg/mL, 1 mg/mL, 0.1 mg/mL (100 μg/mL) and 0.01 mg/mL (10 μg/mL) were 2.4%, 4.3%, 11.4% and 15.7%, respectively. The percentage of cell viability of AgNPs concentrations of 0.1 mg/mL (100 μg/mL), 0.01 mg/mL (10 μg/mL), 0.001 mg/mL (1 μg/mL) and 0.0001 mg/mL (1 μg/mL) on cell lines were found to be 4.2%, 7.3%, 8.7% and 28.1%, respectively. When the results were assessed, the cytotoxic effect of green synthesis AgNPs on MCF-7 cell lines was found to be stronger than *V. opulus* extract. The IC_50_ value of the green synthesis AgNPs is found to be 0.00027 μg/mL (22 ng/mL) for the MCF-7 cell line, and the IC_50_ value of the *V. opulus* fruit extract was 0.021 μg/mL.

Related to AgNPs similar results have been previously reported by other researchers through experimental studies. Several studies reported that AgNPs were shown in the different mechanisms of cytotoxicity, internalization into cancer cells, a cascade of processes starts with loss of inner homeostasis and redox state destabilization [48–51]. In addition, Korkmaz et al., in a study they conducted in 2020, obtained AgNPs synthesized by chemical synthesis in nanotubes and investigated their biological properties [52].

## 4. Conclusion

Today, chemicals and synthetic drugs have been the subject of intense debate due to their effects on human health and the environment. That’s why scientists are looking for new products and new methods that do not have any adverse effects on human health and the environment. In the study, AgNPs were synthesized with the extract obtained from Gilaburu (*V. opulus*) fruits. In addition, the biological (antimicrobial, antibiofilm and anticancer) activities of AgNPs functionalized with natural compounds obtained from *V. opulus* fruit extract and fruit extract were investigated. As a result of the characterization processes, it was determined that the biosynthetic silver nanoparticles (AgNPs) formed the maximum absorbance peak in the 450 nm UV band, and the average particle size was 52.32 nm by X-ray diffraction (XRD) analysis. SEM images of the synthesized AgNPs were taken to determine the particle size and shape of the nanoparticles. AgNPs had different sizes and shapes, ranging from 45 nm to 62 nm (average 52 nm). In addition, it was observed that AgNPs showed a heterogeneous distribution by SEM analysis. The fact that *V. opulus* extract and AgNPs inhibit bacterial growth even at a very low concentration of 0.0312 mg/mL shows that they have a good antibacterial activity. There are few studies in the literature on biofilm inhibition of AgNPs. The present study showed that the biofilm removal effect of biosynthetic AgNPs on *B. subtilis* strain was 86.07% at 1 mg/mL concentration. It also shows that dose-dependent *V. opulus* extract and AgNP applications reduce the growth of MCF-7 cancer cells and have little cytotoxic activity on HUVEC cell lines. The data presented in the present study show that nanoparticles obtained by biosynthesis can be used for the development of antibacterial products in the food industry and for the prevention of biofilm formation in water treatment plants. Moreover, these AgNPs may be an alternative for the control of antibiotic-resistant microorganisms. With the increase in nanotechnological research, it is thought that biosynthesized AgNPs will open a new field in the pharmaceutical industry in the production of pharmaceutical products to make biomedical and industrial products more useful.

## Figures and Tables

**Table 1:** Disk diffusion results of *V. opulus* extract and green synthesis AgNPs (mm).

	Extract	AgNP	TE 10
***Enterobacter aerogenes*** ** ATCC 13048**	7.5 ± 0.0	7.1 ± 0.14	15.6
***Salmonella infantis***	7.1 ± 0.14	7.05 ± 0.07	17.6
***Klebsiella pneumoniae***	8.1 ± 0.14	7.0 ± 0.0	19.7
***Pseudomonas aeruginosa*** ** DSMZ 50071**	8.1 ± 0.14	7.05 ± 0.07	19.7
***Salmonella kentucky***	7.1 ± 0.14	7.0 ± 0.0	18.3
***Enterococcus faecalis*** ** ATCC 29212**	7.6 ± 0.14	7.0 ± 0.0	14.1
***Listeria innocua***	7.3 ± 0.14	7.0 ± 0.0	18.6
***Salmonella enteritidis*** ** ATCC 13075**	8.0 ± 0.0	7.1 ± 0.14	18.0
***Enterococcus durans***	7.6 ± 0.28	7.0 ± 0.0	19.7
***Salmonella typhimurium***	-	-	8.7
***Staphylococcus aureus*** ** ATCC 25923**	-	-	17.3
***Staphylococcus epidermidis*** ** DSMZ 20044**	7.05 ± 0.07	6.05 ± 0.07	8.6
***Bacillus subtilis*** ** DSMZ 1971**	8.15 ± 0.07	6.1 ± 0.14	17.3
***Escherichia coli*** ** CFAI ATCC 25922**	7.1 ± 0.14	7.15 ± 0.21	13.9
***Saratia marrescens*** ** ATCC 13048**	-	-	7.9

(−): There is no inhibition zone. (TE 10): Tetracycline (10 mg/ml).

**Table 2:** MIC results of *V.opulus* extract and green synthesis AgNPs against test microorganisms (mg/mL).

	Extract	AgNP
***Enterobacter aerogenes*** ** ATCC 13048**	25	0.25
***Salmonella infantis***	25	0.25
***Klebsiella pneumoniae***	25	-
***Pseudomonas aeruginosa*** ** DSMZ 50071**	25	0.25
***Salmonella kentucky***	25	0.25
***Enterococcus faecalis*** ** ATCC 29212**	25	0.25
***Listeria innocua***	25	0.25
***Salmonella enteritidis*** ** ATCC 13075**	25	0.25
***Enterococcus durans***	25	-
***Salmonella typhimurium***	25	0.5
***Staphylococcus aureus*** ** ATCC 25923**	25	0.25
***Staphylococcus epidermidis*** ** DSMZ 20044**	25	0.25
***Bacillus subtilis*** ** DSMZ 1971**	25	0.5
***Escherichia coli*** ** CFAI ATCC 25922**	25	0.25
***Saratia marrescens*** ** ATCC 13048**	25	0.25

(−): No inhibition.

**Table 3:** Minimum bactericidal concentrations (mg/mL) of *V. opulus* extract and green synthesis AgNPs that inhibited the growth of test microorganisms by 99.9%.

	Extract	AgNP
***Enterobacter aerogenes*** ** ATCC 13048**	50	0.5
***Salmonella infantis***	50	0.5
***Klebsiella pneumoniae***	50	-
***Pseudomonas aeruginosa*** ** DSMZ 50071**	50	0.5
***Salmonella kentucky***	50	0.5
***Enterococcus faecalis*** ** ATCC 29212**	50	0.5
***Listeria innocua***	50	0.5
***Salmonella enteritidis*** ** ATCC 13075**	50	0.5
***Enterococcus durans***	50	-
***Salmonella typhimurium***	50	1
***Staphylococcus aureus*** ** ATCC 25923**	50	0.5
***Staphylococcus epidermidis*** ** DSMZ 20044**	50	0.5
***Bacillus subtilis*** ** DSMZ 1971**	50	1
***Escherichia coli*** ** CFAI ATCC 25922**	50	0.5
***Saratia marrescens*** ** ATCC 13048**	50	0.5

(−): No inhibition.

**Table 4:** The % inhibition values of the antibiofilm activities of different concentrations (mg/mL) of *V. opulus* extract.

	200	100	50	25	12,5	6,25
***Salmonella kentucky***	58.85	52.25	47.3	39.6	26.85	13.55
***Enterococcus faecalis ATCC 29212***	56.2	45.5	45	24.35	17.8	8.45
***Bacillus subtilis DSMZ 1971***	54.65	46.6	45.5	37.55	27.35	8.8
***Escherichia coli CFAI ATCC 25922***	44.3	34.9	20.05	10.8	5.35	-

(−): No biofilm inhibition.

**Table 5:** The % inhibition values of the antibiofilm activities of different concentrations (mg/mL) of AgNPs obtained from *V. opulus*.

	1	0,5	0,25	0,12	0,06	0,03
***Salmonella kentucky***	77.83	77.44	69.57	65.63	60.33	54.34
***Enterococcus faecalis ATCC 29212***	67.93	62.87	61.02	58.06	49.19	24.74
***Bacillus subtilis DSMZ 1971***	86.07	83.30	77.06	71.42	63.73	35.58
***Escherichia coli CFAI ATCC 25922***	70.35	62.77	62.19	61.58	61.08	48.06
